# How HLA diversity is apportioned: influence of selection and relevance to transplantation

**DOI:** 10.1098/rstb.2020.0420

**Published:** 2022-06-06

**Authors:** André Silva Maróstica, Kelly Nunes, Erick C. Castelli, Nayane S. B. Silva, Bruce S. Weir, Jérôme Goudet, Diogo Meyer

**Affiliations:** ^1^ Departamento de Genética e Biologia Evolutiva, Universidade de São Paulo, São Paulo, SP, Brazil; ^2^ Departamento de Patologia, Universidade Estadual Paulista - Unesp, Faculdade de Medicina de Botucatu, Botucatu, SP, Brazil; ^3^ Molecular Genetics and Bioinformatics Laboratory, Experimental Research Unit, School of Medicine, São Paulo State University - Unesp, Botucatu, SP, Brazil; ^4^ Department of Biostatistics, University of Washington, Seattle, WA 98195, USA; ^5^ Department of Ecology and Evolution, University of Lausanne, 1015 Lausanne, Switzerland; ^6^ Swiss Institute of Bioinformatics, University of Lausanne, 1015 Lausanne, Switzerland

**Keywords:** population structure, MHC, HLA genes, transplantation, population-specific *F*_ST_

## Abstract

In his 1972 paper ‘The apportionment of human diversity’, Lewontin showed that, when averaged over loci, genetic diversity is predominantly attributable to differences among individuals within populations. However, selection can alter the apportionment of diversity of specific genes or genomic regions. We examine genetic diversity at the human leucocyte antigen (HLA) loci, located within the major histocompatibility complex (MHC) region. HLA genes code for proteins that are critical to adaptive immunity and are well-documented targets of balancing selection. The single-nucleotide polymorphisms (SNPs) within HLA genes show strong signatures of balancing selection on large timescales and are broadly shared among populations, displaying low *F*_ST_ values. However, when we analyse haplotypes defined by these SNPs (which define ‘HLA alleles’), we find marked differences in frequencies between geographic regions. These differences are not reflected in the *F*_ST_ values because of the extreme polymorphism at HLA loci, illustrating challenges in interpreting *F*_ST_. Differences in the frequency of HLA alleles among geographic regions are relevant to bone-marrow transplantation, which requires genetic identity at HLA loci between patient and donor. We discuss the case of Brazil's bone marrow registry, where a deficit of enrolled volunteers with African ancestry reduces the chance of finding donors for individuals with an MHC region of African ancestry.

This article is part of the theme issue ‘Celebrating 50 years since Lewontin's apportionment of human diversity’.

## 1. Introduction

In ‘The apportionment of human genetic diversity’, Richard Lewontin addressed a well-defined and answerable question: ‘how much of human diversity between populations is accounted for by more or less conventional racial classification?’ [1, p. 386]. With the genetic data available at the time and drawing on existing classifications available in the anthropological literature, he reached the unequivocal result that individuals assigned to what he heuristically defined as ‘different races’ are, on average, only slightly more genetically different than those from the same ‘race’ (see [2–4] for commentaries on the background and impact of Lewontin's paper). Subsequent studies shifted the focus to an understanding of how human genetic variation is distributed across the globe, the geographic scales at which such variation is observed, and which evolutionary processes account for the observed patterns. What Lewontin referred to as ‘apportionment’ has largely been recast in terms of ‘population structure’, and his approach to describing variation is now explored using metrics related to population genetic models, as is the case of studies using the fixation index (*F*_ST_) [5,6].

Lewontin was aware that his main result referred to an average behaviour over loci, and that variation in population structure among loci arises as a consequence of evolutionary sampling, as well as the locus-specific effects of natural selection. He explored this idea in Lewontin & Krakauer [7] by using the properties of the observed distribution of *F*_ST_ over loci to make inferences about natural selection. While this effort had limited success due to the difficulty in defining an appropriate null expectation for the distribution of *F*_ST_ [8], the strategy has since become a central approach to study natural selection and opened the path to various developments [9–12].

**Figure 1.**
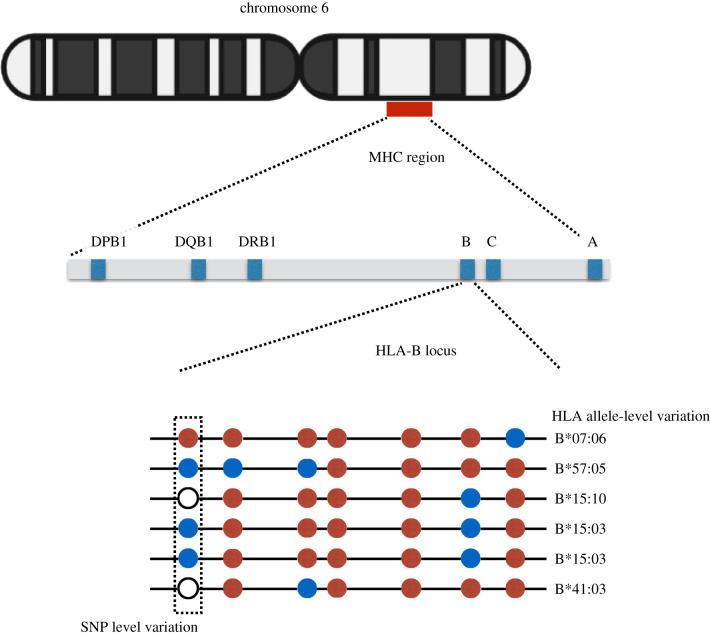
Schematic representation of chromosome 6, the MHC region, location of HLA loci and variation within a specific locus (*HLA-B*). Top: Chromosome 6, showing approximately 4 Mb region comprising the MHC, within which HLA genes are located. Middle: MHC region, with classical HLA class I and HLA class II loci. Bottom: Representation of six sequences of the *HLA-B* coding region, with SNPs represented as circles, with the colour indicating the allele at that position. Analyses of diversity can be carried out over SNPs (with one SNP represented by the vertical rectangle) or over HLA alleles (in this figure, there are five distinct *HLA-B* alleles which are named, among the six chromosomes sampled).

Here, we discuss the apportionment of genetic diversity for a particular set of loci, the human leucocyte antigen (HLA) genes, which are located on chromosome 6 in a region of approximately 4 megabases (Mb) known as the major histocompatibility complex (MHC) (figure 1). The HLA genes have long attracted the interest of evolutionary biologists because of their unusually high levels of polymorphism [13], a pattern originally documented with serological studies and which has been confirmed in many subsequent surveys using molecular techniques [14]. The HLA molecules present fragments of proteins to lymphocytes, allowing a response to be triggered when these fragments are identified as being ‘foreign’ (e.g. when they originate from a pathogen or from a protein altered due to tumorigenesis, see box 1 for more detail on HLA function). The HLA molecules expressed by an individual will therefore define the set of peptides that can be presented and, as a consequence, the set of pathogens which the person can effectively respond to.

Box 1. **HLA function.**

The MHC is an approximately 4 Mb genomic region on chromosome 6 that harbours over 200 loci, most of which are involved in immunity. Among these loci, the human leucocyte antigen (HLA) genes code for proteins that participate in antigen processing and presentation pathways. Classical HLA molecules bind peptides of intra- and extracellular origin and present these on the cell surface to T-cell receptors of T lymphocytes, which initiate an immune response if the peptide bound to the HLA molecule is recognized as being ‘non-self’ [15]. These peptides (or antigens) are protein fragments of 8–12 amino acids originating from degraded cytosolic proteins or internalized proteins. They might be fragments from a microorganism, from a protein altered due to tumorigenesis, or even from normal proteins. The peptides which an HLA molecule can bind are determined by a subset of amino acid positions which define the peptide-binding region of the HLA molecule. As a consequence, different HLA molecules will bind different sets of peptides, and an individual's HLA genotype will determine the repertoire of peptides (and thus the pathogens) that he or she can respond to. This association between HLA genotype and the pathogens underlies evolutionary models that explain HLA polymorphism (see box 2). While the classical HLA class I genes *HLA-A, HLA-B and HLA-C*, code for molecules that bind intracellular peptides and present them to T CD8 lymphocytes (cytotoxic T), the classical HLA class II genes code for molecules that bind mostly exogenous peptides, presenting them to T CD4 lymphocytes (T helper cells). For accessible overviews of HLA biology, see Rock *et al*. [16] and Radwan *et al*. [17].

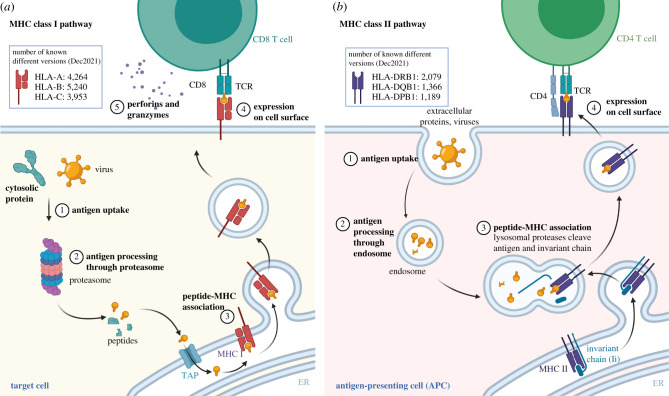

A schematic representation of the HLA class I pathway: the upper left-hand side of each panel shows the total number of different known proteins coded by each locus. (*a*) MHC class I pathway. Proteins originating from intracellular pathogens (1) are processed (2) generating small peptides, which are transported to the endoplasmic reticulum and loaded onto the HLA molecules (3). These are transported to the cell surface and, if the HLA-peptide complex is bound by a T-cell receptor and recognized as non-self, an immune response can be initiated (4). (*b*) MHC class II pathway. Proteins of extracellular origin are endocytosed in vesicles (1), processed within endosomes (2) and cleaved within lysosomes (3), and attached to the surface of a Class II molecule, in the place originally occupied by an invariant chain. The MHC-peptide complex is then transported to the cell surface. Adapted from ‘MHC Class I and II Pathways', by BioRender.com (2022). Retrieved from https://app.biorender.com/biorender-templates.

From an evolutionary perspective, the extreme polymorphism of HLA loci calls for an explanation since neutral processes of mutation and drift cannot account for the observed diversity. The classical explanation for high polymorphism at these loci has been heterozygote advantage, since individuals carrying a broader array of HLA molecules (as is the case of heterozygotes compared to homozygotes) can potentially mount a response to a greater number of pathogens. However, other modes of selection can also result in high levels of diversity (box 2), and it is increasingly plausible that several evolutionary mechanisms need to be invoked to explain the observed polymorphism [17,29].

Box 2. **Evolutionary mechanisms shaping HLA polymorphism.**

Many different ‘tests of neutrality’ show that the extraordinary level of polymorphism of HLA genes cannot be explained by neutral processes of mutation and drift.

— More alleles are found at intermediate frequencies than expected under neutrality, resulting in a deficit of rare alleles [18].
— HLA alleles show an excess of non-synonymous differences [19] and high diversity [20] in the region that codes for amino acids responsible for binding peptides, suggesting that selection has favoured functional changes in the HLA molecules.
— Several polymorphisms within HLA genes are shared between humans and other apes, a pattern that would not be expected under neutrality, given the time depth of speciation [21].
— The nucleotide-level heterozygosity within HLA alleles is skewed to unusually high values with respect to genomewide values [22], resulting in an excess of common variants and a depletion of singletons [23].

Several evolutionary processes can explain the pattern of variation seen at HLA loci.

— Individuals who are heterozygous at an HLA locus can potentially bind more peptides than homozygous individuals, resulting in protection against a broader range of pathogens and therefore higher fitness. Such *heterozygote advantage (*or overdominance) models of selection have a long history in the study of HLA polymorphism [24].
— Selection at HLA genes may be frequency dependent, where common HLA alleles experience a reduction in fitness, since pathogens are recurrently exposed to them, and therefore more likely to be selected for escape mutations, which result in the pathogen not being effectively bound by the HLA molecule, thus reducing its fitness. Low-frequency HLA alleles, on the other hand, are less likely to encounter pathogens which were selected to escape their presentation, and therefore have higher fitness values [25]
— Selection may vary over space or time. For example, in an environment where different regions experience selection for different variants, the overall effect may be the maintenance of polymorphism across the entire species [26]. Likewise, if the pathogen repertoire varies over time and exerts a selective effect on HLA polymorphism, polymorphism can be maintained (even in the absence of heterozygote advantage) [27,28].

These diverse mechanisms fall under the umbrella of forms of ‘balancing selection’, which is defined as a form of selection that results in the maintenance of higher levels of diversity than would be expected under neutrality.

The extreme polymorphism of HLA genes is also relevant to medical practice. For example, variation at HLA loci explains differences among people in the susceptibility and resistance to infectious diseases [30], the prognosis for cancer treatment [31] and susceptibility to autoimmune conditions [32]. An understanding of HLA diversity is also relevant to haematopoietic stem cell transplantation (HSCT, often referred to as bone marrow transplantation), an important curative procedure used in the treatment of various forms of cancer and haematological diseases, where cells capable of generating haematopoietic tissue are transferred from a donor to a recipient. The ideal setting for HSCT is that of identity for at least five HLA loci between patient and donor. A question that naturally arises is whether the chances of finding an HLA match are greater among individuals with greater shared ancestry (for example, because their ancestry traces to the same continent). This question is directly connected with the central theme of Lewontin's [1] paper and provides a concrete example of how an understanding of the apportionment of variation at a specific set of loci can have consequences for medical practice.

In this paper, we will explore two interconnected issues: how genetic diversity at HLA loci is apportioned and the consequences of such apportionment for bone-marrow transplantation. We use the 1000 Genomes Project data [33,34] to illustrate the patterns found for HLA loci and the MHC region, as well as the challenges of interpreting the measures of apportionment when applied to extremely polymorphic loci. We then discuss how the observed apportionment of variation provides information about the chance of finding compatible donors sampled from the same or different geographic regions. We illustrate our discussion with examples from Brazil, a country with a highly admixed population, with large components of European, African and Native American ancestry [35,36]. We conclude with a discussion regarding the benefits and caveats of grouping individuals into categories in the process of recruiting donors for bone-marrow transplantation.

## 2. How is human leucocyte antigen diversity apportioned?

To investigate the apportionment of genetic diversity of HLA genes, we analyse sequence-level data for 20 populations, grouped into four geographic regions, and use *F*_ST_ as a metric to describe the relative degree of within and among group kinship (see §5). We note that there is no single correct way to define what constitutes a ‘locus’ to be used in analyses of the apportionment of genetic diversity. We have chosen to analyse the same data using two definitions of a genetic locus: (i) at the level of SNPs, where sites are treated individually and then averaged; (ii) using a combination of non-synonymous SNPs within the coding region for an HLA gene to define an ‘HLA allele’. Under this definition, each ‘HLA allele’ corresponds to a unique HLA protein (figure 1).

An objective way to compare the apportionment of variation at HLA genes with genomewide values is to contrast *F*_ST_ estimates for SNPs within HLA genes to genomewide values (thus controlling for marker types), using the same set of populations and individuals (thus controlling for population and individual sampling effects). We use population-specific *F*_ST_ [37] to make inferences about population structure. Whereas standard *F*_ST_ provides a single estimate of the mean coancestry within populations relative to the mean coancestry between populations, population-specific *F*_ST_ estimates each population's mean coancestry relative to the mean coancestry between populations. Standard *F*_ST_ is the average of population-specific *F*_ST_ values.

**Figure 2.**
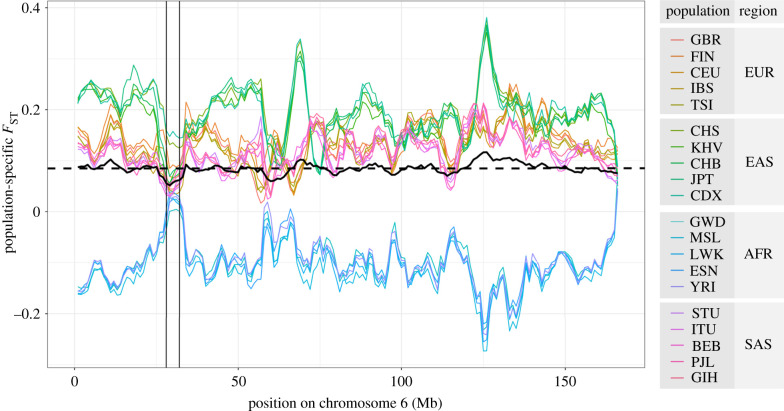
Population-specific *F*_ST_ across chromosome 6, in overlapping windows of 5 Mb, with a step-size of 1 Mb. The *F*_ST_ values were estimated for each population relative to all other populations available (i.e. each population relative to the entire world, with no geographic grouping used). Each coloured line represents a specific population; the black line represents the overall *F*_ST_ for each window; the black dashed line is the average overall *F*_ST_ for the entire chromosome. The vertical lines delimit the MHC region, within which HLA genes are contained. See electronic supplementary material, table S1, for 1000 Genomes abbreviations of population and region names.

To visualize how population structure in the MHC region compares to genomewide values, we estimated the population-specific *F*_ST_ for 5 Mb windows (1 Mb step-size) along chromosome 6 (figure 2). The five African populations have lower population-specific *F*_ST_ throughout the entirety of chromosome 6, reflecting higher within-population diversity and thus lower within-population kinship among individuals, as compared to kinship within populations from other regions. However, within the MHC region, Africans have a higher population-specific *F*_ST_ than in the remainder of the chromosome, and Asian and European populations show the opposite, i.e. a reduction in their *F*_ST_ values within the MHC (figure 2, see MHC region delimited by vertical lines). The increase seen for *F*_ST_ in the African MHC region reflects the fact that, for this genomic region, Africans do not have a markedly higher diversity (and thus a lower degree of within-population kinship) than do other populations. The overall *F*_ST_ (i.e. the average of population-specific *F*_ST_ values) reaches its lowest value in chromosome 6 within the MHC (black line in figure 2). Although the window size used in figure 2 (5 Mb) is much larger than the region influenced by balancing selection, the analysis illustrates how variation within a small set of loci shapes features computed for a much larger region (explaining why the MHC region as a whole is often filtered out when genomewide inferences of demographic history are of interest).

**Figure 3.**
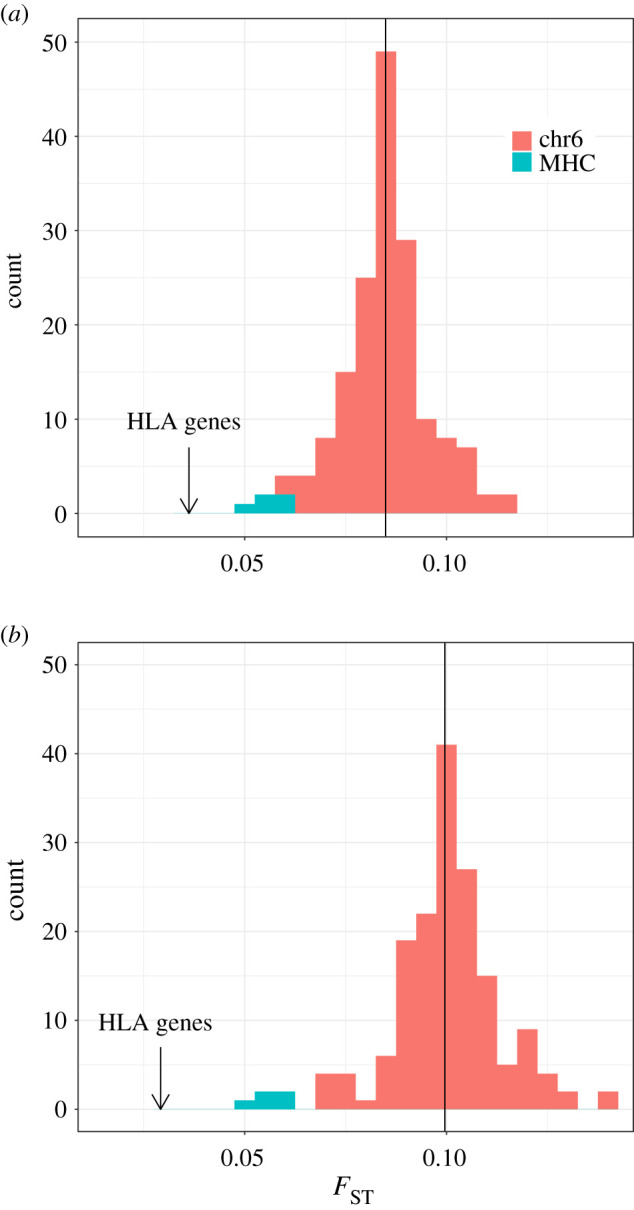
Distribution of overall *F*_ST_ for 168 windows along chromosome 6. The *F*_ST_ values for windows that have at least 2 Mb contained within the MHC are shown in green, while the remaining windows on chromosome 6 are shown in red. The arrow indicates the overall *F*_ST_ for the SNPs contained within the three HLA loci we studied (*HLA-A, -B, -C*), and the vertical line is the average *F*_ST_ over all windows. (*a*) Overall *F*_ST_ among populations, where the kinship within each population is compared to that among populations, regardless of the continent the population belongs to. (*b*) Overall *F*_ST_ among continents, where all populations within a continent were merged and treated as a single group, and *F*_ST_ compares kinship within continents to that among continents.

The outlier status of the windows in the MHC region can be visualized by comparing the distribution of their *F*_ST_ values to that of windows in the remainder of chromosome 6 (figure 3*a*). The overall *F*_ST_ for the set of SNPs contained within the three HLA genes we analysed (a subset of the MHC region) is even more extreme (figure 3*a*, vertical arrow). The apportionment of diversity among regions can also be computed by treating all the populations in a geographic region as a single group and computing an overall *F*_ST_ among regions (figure 3*b*). Once again, the *F*_ST_ values for SNPs within the MHC and HLA genes occupy an extreme position, indicating unusually low *F*_ST_ with respect to the remainder of chromosome 6 (with similar results obtained in analyses using windows of 1Mb; see electronic supplementary material).

In their study of the effects of selection on *F*_ST_, Lewontin & Krakauer [7, p. 187] proposed an interpretation for patterns of low *F*_ST_, such as those we describe for the MHC region and for HLA genes: ‘it is among the gene frequencies that have not diverged, those associated with small F values, that we should look for selection. In particular we should look for heterotic selection tending to retard divergence among the isolated groups with respect to these loci.’

While the analysis of individual SNPs provides a compelling case that *F*_ST_ values within the MHC region and HLA genes are lower than the rest of chromosome 6, it is the HLA protein, defined by a combination of amino acids (figure 1), which has properties that determine the peptides that can be bound and the degree of identity between donor and recipient, in the context of bone-marrow transplantation. We therefore re-coded the 1000 Genomes data for *HLA-A*, *-B* and *-C* loci at the level of HLA alleles. To quantify the apportionment of genetic diversity, we revisited Lewontin's approach, applying it to 20 human populations, divided into four continental groups. In line with the results of Lewontin [1], we found that for the 1000 Genomes data most of the variation lies within populations (table 1, first row). We also revisited previous studies which specifically targeted the apportionment of variation at HLA loci (table 1, last three rows). While these studies are not directly comparable to each other due to different sampling designs (number of populations and continents) and HLA loci included, in all cases the majority of HLA variation lies within populations, and a small fraction of the total variation is attributable to differences among continental groups of populations. Notice that this does not imply that there are few differences between populations and geographic regions, but rather that they add very little diversity relative to that which is already present within populations.

**Table 1.** Apportionment of diversity at the level of HLA alleles, in different studies. The first row presents our results computed using Lewontin's apportionment of variation framework, comparing measures of diversity for populations, continental regions and the species as a whole. All other rows report results from studies that use *F*_ST_-based metrics to estimate the within- and among-population diversity. The present study surveyed *HLA-A*, *-B* and *-C*; Meyer *et al.* [38] and Sanchez-Mazas [39] surveyed *HLA-A*, *-B*, *-C*, -*DRB1* and -*DQB1*; Ryman *et al*. [40] surveyed *HLA-A* and *-B*.

within populations	among populations	study
95%	5%	present analysis
91%	9%	[39]
90%	10%	[38]
87%	13%	[40]

Low *F*_ST_ values are commonly interpreted as indicating extensive sharing of alleles among populations. While this interpretation is in general appropriate for the analysis of predominantly biallelic SNP (figures 2 and 3), for highly polymorphic multi-allelic loci low *F*_ST_ values can be found even when there are few (or even no) shared alleles among populations [41,42]. The analysis of Alcala and Rosenberg [43] highlights this, by mathematically exploring constraints on *F*_ST_, and demonstrating that high mutation rates decrease the frequency of the most frequent allele in a multi-allelic locus, and thus leads to lower *F*_ST_ values. Although the mutation rate at individual sites within the MHC does not exceed genomewide averages, the high density of non-synonymous polymorphism results in an extremely large number of alleles present in our species (see box 1), an effect analogous to a high mutation rate at the allele level. This must be accounted for when interpreting the low *F*_ST_ values found for HLA loci (table 1). A similar challenge occurs in the forensic genetics literature, where markers with high heterozygosity (chosen for their potential for individual identifiability) show low *F*_ST_, but provide high population identifiability, again demonstrating the challenge of interpreting low *F*_ST_ values in the context of highly polymorphic loci [44]. In the next section, we discuss the connections between interpretations of how HLA diversity is apportioned and success in locating compatible donors for bone-marrow transplantation.

## 3. Human leucocyte antigen diversity and bone-marrow transplantation

Patients suffering from haematological diseases, including forms of cancer, can be treated with stem cells harvested from the bone marrow of a donor in a process called haematopoietic stem cell transplantation (HSCT). The ideal setting for this procedure is for donor and recipient to match both alleles at five HLA loci (*HLA-A*, *-B*, *-C*, *-DRB1*, *-DQB1*, referred to as 10/10 matching), with ‘matching’ defined by identity at the protein level for HLA alleles. However, because the expected heterozygosities at the HLA allele level are typically greater than 0.90 within populations [14], the chance that two unrelated individuals will match at five loci is extremely low. Therefore, the first option for patients is to search for compatible donors among close relatives. However, as exemplified by an analysis of the USA National Marrow Donor Program (NMDP), fewer than 30% of patients find a 10/10 match among relatives [45], and their option is to seek donors in registries.

Given the importance of registries in locating potential donors, maximizing the effectiveness of recruitment of donors is an important challenge. In this context, a question which arises is whether genetic ancestry of the patient and the donor influences the chance of a match. The population structure of HLA loci is potentially informative about this question. For example, in a setting in which populations have markedly different repertoires of HLA alleles, finding matching individuals from different populations will be less likely than from within the same population. We explore the relationship between population structure and matching of HLA alleles for transplantation in the Brazilian population. Brazil is a highly admixed country, with the largest population descended from Africans outside Africa [46], a consequence of the forced displacement of over 4 million Africans, taken to Brazil and enslaved. A review of over 51 studies estimated that the mean African ancestry in Brazil is approximately 20% and is close to 30% in the country's northeast [36]. Native American genetic ancestry is also present, but lower than that of many other South American countries, and was estimated to be approximately 10%, reaching up to 27% in the north of the country [36].

**Figure 4.**
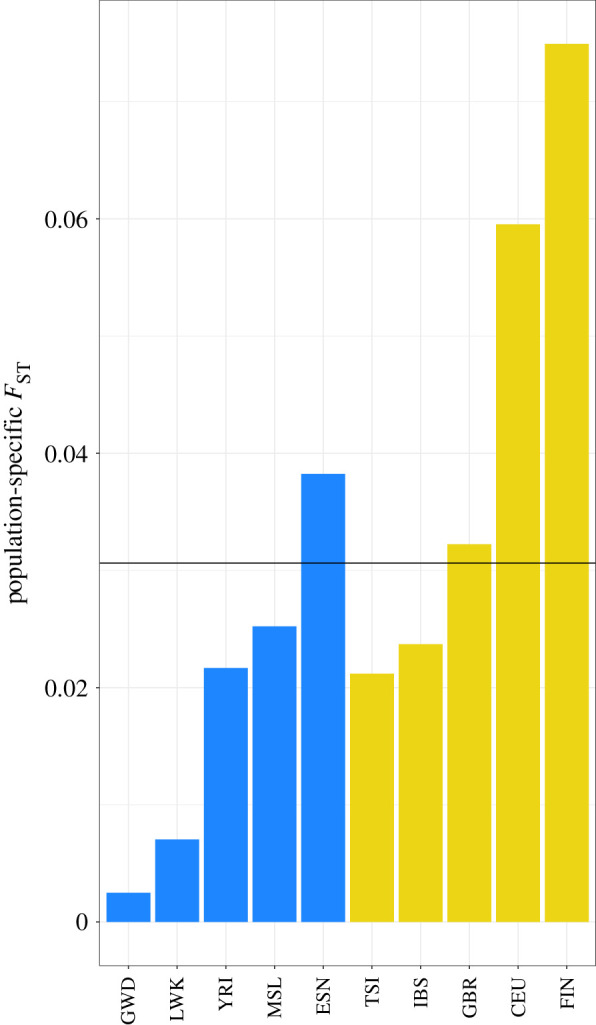
The population-specific *F*_ST_ values for African (in blue) and European (in yellow) populations in the 1000 Genomes data, estimated for diversity at the level of HLA alleles for *HLA-A, -B* and *-C*. The horizontal line is the average population-specific *F*_ST_, with a value of 0.03. Each population-specific *F*_ST_ was estimated by comparing the allele sharing within each population to that for the entire dataset, comprising the five African and five European populations.

For the two most common ancestries in Brazil (European and African), we computed the population-specific and overall *F*_ST_ for populations in the 1000 Genomes data and found values to be very low (figure 4, overall *F*_ST_ = 0.03 at the HLA allele level). At first sight, this appears to indicate that there is an extensive sharing of alleles among Africans and Europeans. However, as discussed previously, for extremely polymorphic loci *F*_ST_ may be low even in the absence of extensive sharing of alleles among populations, making it misleading to associate low *F*_ST_ to lack of differentiation.

**Figure 5.**
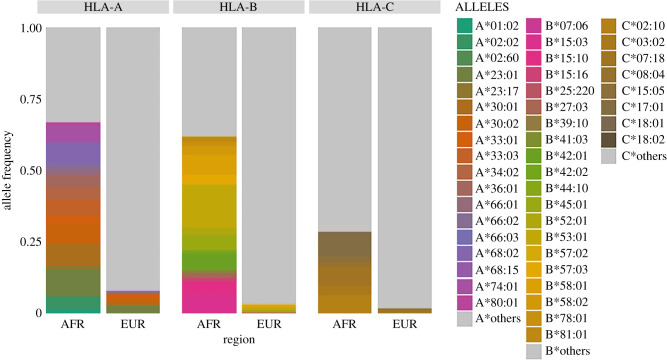
Frequencies of a subset of HLA alleles for African and European populations (1000 Genomes data). To visualize the differences in allele frequencies between regions, we selected alleles that were either exclusive to Africa or found at frequencies that were at least threefold greater in Africa than Europe. We identify each allele by a distinct colour. ‘Others’ (in grey) refers to the cumulative frequency of all alleles that are either exclusively found in Europe, or that do not occur threefold more frequently in Africa than Europe, or that are shared between them.

How extensive are the differences in allele frequencies among Europeans and Africans, for the 1000 Genomes data? A simple way to address this question is to directly visualize the differences in allele frequencies among regions. We identified a set of HLA alleles that are either exclusive or at least threefold more common in Africa, as compared to Europe, in the 1000 Genomes data, and compared their contribution to the frequencies in these regions (figure 5).

We see that 18 *HLA-A* and 20 *HLA-B* alleles, which collectively contribute to approximately 60% cumulative frequency in Africa, reach less than 10% in Europe (for *HLA-C* alleles the differences among regions are smaller). The striking differences in allele frequencies among geographic regions for *HLA-A* and *HLA-B* were also observed in an independent dataset containing data from multiple populations [14] (electronic supplementary material, figures S1–S3). The allele frequencies in figure 5 show that the distribution of HLA alleles is in fact geographically structured, but as a consequence of the extreme polymorphism the overall *F*_ST_ remains low [41,42]. We do not see this as a limitation of *F*_ST_, but as an expression of the information *F*_ST_ conveys, which is related to the evolutionary history of the population and locus of interest, rather than serving as a direct measure of differences in allele frequencies. Here, low *F*_ST_ reflects the high diversity (and low kinship) both within and between regions, a key feature of HLA polymorphism.

**Figure 6.**
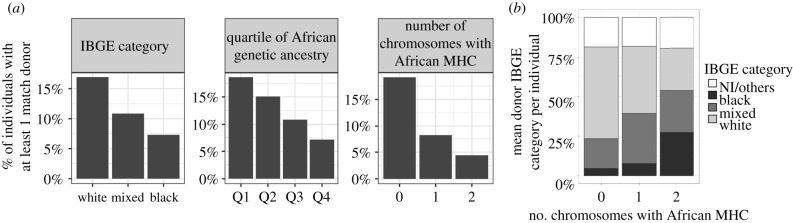
Ancestry and donor availability in the Brazilian bone-marrow registry (REDOME, *Registro Nacional de Doadores Voluntários de Medula Óssea*) for matching at both alleles at five loci (10/10 matching). (*a*) Admixed individuals were classified following three possible criteria: self-assigned ‘IBGE category’ (defined by the *Instituto Brasileiro de Geografia e Estatística*, the Brazilian Institute of Geography and Statistics); genomewide African ancestry, divided into quartiles; number of *F*_ST_ chromosomes for which the MHC is African (0, 1 or 2). The *y*-axis represents the proportion of individuals with at least one compatible donor. (*b*) The self-assigned identifier (a proxy coarsely related to ancestry) of potential donors, with respect to the genetic ancestry in the MHC region of individuals seeking donors. Notice that for individuals seeking donors and who carry two African chromosomes in the MHC region, ‘Black’ and ‘Mixed’ make up the largest fraction of potential donors (adapted from Nunes *et al.* [47]).

What are the consequences of the large differences in allele frequencies between geographic regions (figure 5) to the chances of finding a matching donor in a repository? Nunes *et al*. [47] quantified how an individual's ancestry influences their chances of finding a matching donor in REDOME, the Brazilian bone-marrow donor registry. Surveying over 8000 admixed individuals, they showed that those who self-identify as ‘Black’ have a reduction in their chances of finding a matching donor, with respect to those who self-identify as ‘White’. Nunes *et al*. [47] also explored the effects of two forms of identifying the individual's genetic ancestry: genomewide ancestry and ancestry specific to the MHC region. In both cases, greater African ancestry was associated with decreased chances of finding a match in REDOME (figure 6). The reduction in chances of finding a match increases as we go from grouping individuals as ‘Black’, to ‘most African genomewide’, and finally ‘African within the MHC region’ [47]. This underscores the fact that, in admixed populations, the decreased chances of finding a match for individuals who identify as ‘Black’ is because this category is serving as a proxy for ancestry within the MHC. A study of the NMDP, which is the registry of volunteer haematopoietic cell donors in the United States, also found that individuals who self-identify as ‘Black’ have a lower chance of finding a donor, compared to those who identify as ‘White’ [45], but did not explore effects of genetic ancestry.

Two factors can explain the lower rates at which individuals with greater African ancestry find matching donors in Brazil's REDOME. First, whereas the categories ‘Black’ and ‘Mixed’ correspond to 54% of Brazilians, they make up only 31% of registered donors in REDOME. Thus, given that African HLA alleles are more common among ‘Black’ and ‘Mixed’ individuals than among those who self-identify as ‘White’ [47], the underrepresentation of the categories enriched with African ancestry contributes to the lower rates at which matches are found. Reduced matching may also be driven by higher diversity in HLA genes of individuals with greater African ancestry. This expectation is supported by the fact that African populations are more diverse than Europeans at *HLA-A* [14,38] (although, for other HLA loci, heterozygosity is surprisingly similar among populations from Africa and Europe). In addition, overall linkage disequilibrium within the MHC region is on average lower among African populations compared to others [48]. Given that matching for transplantation requires identity over multiple HLA loci, the higher haplotypic diversity among Africans further contributes to the difficulty in finding matching donors.

Would increasing the proportion of individuals with African ancestry in the registries increase the chances of patients with a greater African ancestry finding a compatible donor? Nunes *et al*. [47] showed that individuals with a higher proportion of African ancestry in the MHC on average find proportionally more donors identified as ‘Black’ or ‘Mixed’ (figure 6*b*). This suggests that, for individuals with greater African ancestry, an increase in the proportion of donors with African ancestry will contribute to their chances of finding a compatible donor. As a consequence, they used their findings to recommend that recruitment of new donors for REDOME be targeted to regions of Brazil where African ancestry is higher (e.g. the northeast of the country) in an effort to reduce the disparity in access to compatible donors. Nunes *et al*.'s [47] analysis of ancestry and matching underscores the importance of genetic diversity in medical research and public policies, and points out the importance of finding an appropriate way to describe HLA diversity.

It may seem surprising to refer to ‘Black’ and ‘Mixed’ when celebrating Lewontin's [1] paper, which provided a categorical rejection of racial categories. Our results show that in the case of finding compatible donors, the government-defined labels are only useful to the extent that they are correlated with genomewide ancestry which, in turn, is correlated with ancestry in the MHC [47]. It is ultimately the genetic ancestry of the MHC which defines the chances of finding a match. Because genetic data are not readily available for the population as a whole, the government-defined labels can provide a proxy for ancestry that can guide recruitment policies. Despite the utility of these labels to the practical problem of transplantation, their continued use can contribute to reifying the incorrect notion that groups such as ‘Black’ and ‘Mixed’ have a well-defined biological identity [49]. This calls for efforts to clearly communicate the difference between labels that have heuristic value for donor recruitment strategies and the existence of meaningful biological groups.

## 4. Discussion

How does the apportionment of genetic variation at HLA loci, which we have discussed in this paper, interface with Lewontin's 1972 paper [1]? Using genomic data, we have shown that SNPs within the MHC region, and specifically those within HLA genes, show unusually low *F*_ST_ values among geographic regions. When we examine the apportionment of variation at HLA alleles (defined by the combination of coding SNPs within each locus), we find a pattern similar to that reported by Lewontin, with the bulk of variation being attributable to differences among individuals within populations and not due to differences among populations. Taken at face value, these results appear to support a ‘lack of population structure’ for HLA diversity. This is, however, a misleading interpretation: the repertoire of HLA alleles present in populations from different regions is in fact quite different (e.g. figure 5), and the reason why *F*_ST_ is low is because within-population diversity is extremely high, constraining the maximum *F*_ST_ value that can be reached [43]. Thus, in the case of HLA genes, equating ‘low *F*_ST_’ to ‘shared diversity’ is inappropriate. In fact, HLA diversity has historically proved highly informative for tracking the movement of human populations for the very reason that many HLA alleles are geographically restricted [50].

Do these findings for HLA genes counter the central message contained in Lewontin [1]? Given Lewontin was clear that his findings referred to an average over many loci, and later explicitly stated that genes with marked frequency differences among populations may exist, but ‘are not typical of the human genome in general’ [51], we believe that the features of HLA polymorphism differ from the average pattern he described, but do not represent a rejection of the main message his 1972 paper conveyed.

However, our discussion also shows that the quantitative findings for HLA based on *F*_ST_ or Lewontin's apportionment approach (e.g. table 1) are not particularly informative about important features of HLA diversity. For example, the simple observation that allele frequencies differ markedly across populations has important practical consequences to our understanding of selective pressures on HLA alleles and to policy decisions involving transplantation.

Lewontin's writings on evolutionary biology recurrently emphasized the challenge of delimiting units of analysis, both at the morphological and genetic levels [52]. Indeed, there is no single correct way to define genotypes for the population structure analyses. We have shown that results for the diversity of SNPs and HLA alleles are both informative, but convey different types of information. The decision with respect to which definition of ‘HLA genotype’ will be used has a bearing on the distribution of genetic variation.

Lewontin's work also emphasized the context dependency of evolutionary trajectories, with empirical and analytical explorations of fitness surfaces that arise when interactions among loci and changing environments are considered [53]. The study of HLA genes provides an example of how a locus can carry the signatures of distinct selective regimes, at different timescales: strong evidence of balancing selection at the level of SNPs, when long timescales are considered, and evidence of recent selection, favouring locally adapted HLA alleles, at recent timescales (involving divergence between populations inhabiting the same continent, or recently admixed populations; see box 3).

Box 3. **Local adaptation at HLA loci.**

The overall *F*_ST_ for a locus captures the influence of evolutionary sampling and natural selection, averaged over populations. However, it is possible that selection acts in a population-specific manner, with the overall pattern masking signatures within specific populations. This has proved a relevant issue for studies of MHC diversity, which have recently found examples of specific populations with signatures of increased *F*_ST_, in the opposite direction to the overall *F*_ST_ values presented above.

For a set of closely related African populations (African-American, Nigerians and Gambians), Bhatia *et al*. [54] found that the *F*_ST_ in the MHC region significantly exceeded that of the rest of the genome. For another set of African populations, Patin *et al*. [55] also found an excess differentiation at the MHC region for Bantu speakers, relative to genomewide. In an analysis of Native Americans, Nunes *et al*. [56] used microsatellites to show that *F*_ST_ in the MHC exceeded neutral expectations. Finally, Brandt *et al*. [57] found that pairwise contrasts between East Asian populations from the 1000 Genomes data showed unusually high *F*_ST_, when compared to genomewide averages.

Thus, while there is strong evidence of reduced overall *F*_ST_ for SNPs in the MHC region and HLA genes (figures 2 and 3 of the manuscript), studies that queried closely related sets of populations identified instances where *F*_ST_ for markers within the MHC region is in fact higher, and not lower, than genomewide averages. These findings are consistent with selection favouring locally adapted variants and emphasize the importance of considering that selective regimes at HLA loci may differ, depending on the timescale being considered [29,58].

Finally, Lewontin was explicit about his political views and assumed that scientists' technical work reflected their inevitable (although not always stated) political perspectives. The study of HLA population structure reveals how a technical question, regarding the degree of population structure in a genomic region, can have a bearing on public health issues in admixed populations. We ourselves were stimulated to investigate the effects of ancestry on the chances of finding donors by both recent scientific work in the United States [45] and by websites maintained by patient-driven organizations (for example, https://blackbonemarrow.com/ and https://bonemarrowwish.org/). In the case of Brazil, the long history of slavery lies at the root of a pattern of systemic racism [59] and has resulted in a social organization where individuals of African ancestry have reduced access to quality healthcare. In addition, there are specific diseases which overburden individuals of African ancestry, such as sickle cell disease [60], for which bone-marrow transplantation is a possible treatment. This calls for, among other efforts, research with the potential to guide institutional healthcare strategies. In the specific case of the Brazilian registry, strategies to recruit donors of African ancestry are a direct recommendation of the genetic analyses.

## 5. Data analysis

### (a) Analysis of chromosome 6

For our reanalysis of population structure in the MHC region, we have chosen Phase 3 of the 1000 Genomes Dataset (hereafter 1000G) [33]. This dataset is appropriate for our questions because it is typed for 20 non-admixed populations from four different geographic regions and has available Sanger sequencing for a subset of HLA genes, allowing us to validate our HLA calls obtained with our in-house HLA calling pipeline implemented via hla-mapper software [61]. We used VCFtools v. 0.1.15 [62] to filter for biallelic SNPs and indels. We filtered out sites deviating from Hardy–Weinberg Expectations (HWE) in at least two populations within any region. We tested for deviation from HWE using an exact test [63] implemented in VCFtools v. 0.1.15 [62] (significance set at *p* < 10^−8^). Testing for deviation from HWE identifies putatively mismapped regions since these are more likely to arise in highly polymorphic regions with low coverage data [64]. We converted genotypic data to dosage format using Plink v.1.9 [65].

The 1000G data allows us to compare the variation of the MHC to that in other genomic regions, make calls for nucleotide positions within HLA loci and estimate which HLA alleles an individual carries. By ‘HLA alleles’, we refer to the phased combination of variants within a locus, an important unit of analysis since HLA alleles define immunological phenotypes that contribute to disease and adaptation. Although the sampling structure of the 1000G is much coarser than that used by Lewontin [1], four major geographic regions are represented (Africa, Europe, South Asia and East Asia). The regions (and populations, followed by their abbreviations) are: Africa (Gambian Mandinka (GWD), Mende (MSL), Esan (ESN), Yoruba (YRI), Luhya (LWK)); Europe (Finnish (FIN), Iberian (IBS), Toscani (TSI), Northern Europeans living in the United States (CEU), British (GBR)); East Asia (Southern Han Chinese (CHS), Kinh Vietnamese (KHV), Japanese (JPT), Han Chinese (CHB), Dai Chinese(CDX)); and South Asia (Bengali (BEB), Punjabi (PJL), Gujarati (GIH), Tamil (STU) and Telugu (ITU)).

### (b) Human leucocyte antigen allele and SNP calls

Publicly available genotype calls for SNPs within HLA loci for the 1000G data show high error rates, a consequence of mapping short reads from an extremely polymorphic region to a single human genome [66]. We therefore made new calls by extracting reads that either map to the MHC region or are unmapped (using the 1000G release described by Byrska-Bishop *et al.* [34]) and then mapping them using known HLA alleles as references [61]. Throughout the text, we refer both to analyses for SNPs within HLA genes (in our case, the *HLA-A*, *-B* and *-C*) and the MHC region (the broader region within which these and other genes of immunological function are contained). We also analyse diversity for ‘HLA alleles’, the phased combination of SNPs that defines a unique HLA protein sequence. We analysed diversity among HLA alleles that correspond to unique protein sequences, given our interest in functional variation at the HLA loci.

### (c) *F*_ST_ estimates

In addition to revisiting Lewontin's approach to quantifying the apportionment of genetic variation [1], we estimate *F*_ST_ to describe population structure. Our analysis uses the framework of Weir & Goudet [37], where population-specific *F*_ST_ is computed based on allelic sharing and interpreted as a measure of relative kinship (see also [11] for other strategies to estimate population-specific *F*_ST_ values).

For genotypic data, for population *i*, we define the population-specific *F*_ST_ metric as

$$F_{\mathrm{ST}}^{i}=\;\frac{S_{w}^{i}-S_{b}}{1-\;S_{b}}$$

The value of S refers to the proportion of allele pairs, one allele from each individual, that are of the same type. For a bi-allelic locus, S assumes a value of one if the two individuals are homozygous for the same allele, zero if they are homozygous for different alleles, and 1/2 otherwise. Multi-allelic loci can be accommodated in this framework by recoding them as k biallelic markers, one for each of k alleles. $S_{w}^{i}$ is sharing within population *i*, and *S_b_* is sharing between pairs of populations, averaged over pairs. Population-specific $F_{\mathrm{ST}}^{i}$ estimates the average kinship within population *i* relative to the average kinship between all other populations [37].

Averaging $F_{\mathrm{ST}}^{i}$ over populations provides the ‘overall *F*_ST_’. When estimating *F*_ST_ for a genomic region containing multiple SNPs, we use the ‘ratio of averages’ approach, where the average value of the denominator and numerator are computed separately over SNPs, providing an unbiased estimator of *F*_ST_ [37,67].

For all analyses, the *F*_ST_ estimates were computed with the Hierfstat R package [68]. In analyses of HLA alleles and SNPs within HLA loci, we used the genind2hierfstat and loci2genind functions (from the Hierfstat and Pegas packages) to format the data, and the fstat2dos and fs.dosage functions (from the Hierfstat package) to convert genotypic data to dosage and then calculate *F*_ST_.

When estimating *F*_ST_ values along chromosome 6, we used sliding windows of 5 Mb and 1 Mb (see electronic supplementary material). These are both substantially larger than the scale at which balancing selection is expected to leave a footprint [69] and can provide information about the contribution of several HLA loci to the overall population structure of the genomic region. We also compute an average *F*_ST_ for the set of SNPs contained within the *HLA-A*, *-B* and *-C* genes themselves, i.e. directly within the loci under balancing selection (represented by the arrow in figure 3). This provides an estimate of *F*_ST_ within a narrowly defined region under selection, complementing the broad pattern captured by the 5 Mb window.

## Data Availability

The primary data we used are publicly available (from the 1000 Genomes resources), as are the programs used for processing. We processed the 1000 Genomes raw data using hla-mapper to obtain calls with increased accuracy. The resulting allele frequencies for each population, for both HLA alleles and SNPs, are available at the ‘data and software’ tab in https://genevol.ib.usp.br.
